# Predicting ICU in-hospital mortality from text-encoded structured EHR data using adaptive transformer layer fusion

**DOI:** 10.1016/j.isci.2026.116135

**Published:** 2026-06-04

**Authors:** Han Wang, Guoguang Lao, Ruoyun He, Ting Liu, Hejiao Luo, Changqi Qin, Hongying Luo, Yingqi Liu, Junmin Huang, Zihan Wei, Lu Chen, Yongzhi Xu, Ziqian Bi, Junhao Song, Tianyang Wang, Xin Liang Chia, Xuanhe Hou, Huafeng Liu, Junfeng Hao, Chunjie Tian

**Affiliations:** 1 Department of Otorhinolaryngology, Affiliated Hospital of Guangdong Medical University, Zhanjiang 524000, China; 2 First Clinical College, Guangdong Medical University, Zhanjiang, China; 3 Guangdong Provincial Key Laboratory of Autophagy and Major Chronic Non-communicable Diseases, Institute of Nephrology, Affiliated Hospital of Guangdong Medical University, Zhanjiang 524001, China; 4 The First Dongguan Affiliated Hospital, Guangdong Medical University, Dongguan 523710, China; 5 Department of Critical Care Medicine, Affiliated Hospital of Guangdong Medical University, Zhanjiang, China; 6 CICU, The Seventh Affiliated Hospital, Sun Yat-sen University, Shenzhen, China; 7 Beijing University of Technology, Beijing 100124, China; 8 China Agricultural University, Beijing 100083, China; 9 Xi’an Jiaotong-Liverpool University, Suzhou 215123, China; 10 JTB Technology Corp., Tainan 741, Taiwan; 11 Department of Family Medicine, Shengjing Hospital of China Medical University, Shenyang 110022, China; 12 Department of Radiation Oncology (MAASTRO), GROW - Research Institute for Oncology and Reproduction, Maastricht University, Maastricht, the Netherlands

**Keywords:** Health sciences, Medicine, Medical specialty, Internal medicine, Intensive care medicine

## Abstract

Early identification of ICU patients at high mortality risk is essential for triage and timely intervention. We present adaptive layer fusion with intelligent attention (ALFIA), a modular architecture that jointly trains low-rank adaptation (LoRA) adapters and an adaptive layer-weighting mechanism to fuse multi-layer semantic features from a pretrained transformer backbone. ALFIA operates on *text-encoded representations of the structured EHR data*, in which tabular clinical variables (demographics, vital signs, laboratory values, and severity scores) are converted into standardized natural-language descriptions rather than processed as free-text clinical notes. Evaluated on the CriticalWindow-24 benchmark with MIMIC-IV and eICU cohorts, ALFIA achieves strong AUPRC while maintaining a balanced precision-recall profile. The learned embeddings can be further combined with gradient boosting (ALFIA-boost) or neural networks (ALFIA-nn) for additional gains. These findings demonstrate that text-encoded structured EHR data can support practical, generalizable early-warning models for ICU mortality risk stratification.

## Highlights

• Adaptive fusion of multi-layer BERT representations improves clinical prediction
• Text-encoded structured EHR leverages semantic relationships from tabular clinical variables
• Achieves state-of-the-art AUPRC (0.585) on ICU mortality prediction, outperforming baselines
• Modular design integrates with GBDTs and neural networks for enhanced performance

## Graphical abstract

**Figure:**
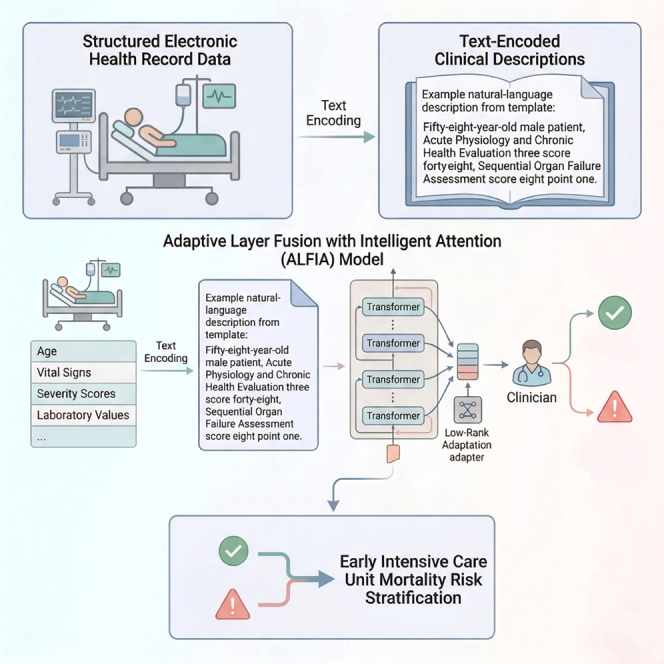

## Introduction

Early identification of in-hospital mortality risk remains a central challenge in intensive care units (ICUs), where clinicians must monitor many unstable patients under severe time pressure. ICU mortality rates remain substantial worldwide, and delayed recognition of deterioration can directly affect triage, escalation of care, and communication with families.^1^^,^^2^ Therefore, accurate risk stratification within the first 24 h of admission is clinically important rather than merely statistically convenient.

Traditional severity scores such as APACHE, SAPS, and SOFA provide a useful foundation but have well-recognized limitations. They summarize patient status with relatively fixed handcrafted rules, rely on limited time windows, and may not fully capture the complex interactions among demographics, vital signs, laboratory results, and severity indicators.^3^^,^^4^^,^^5^^,^^6^ Recent ICU prediction studies, including 2024–2025 work on enhanced severity modeling and causal deep-learning frameworks, show that machine learning can improve forecasting, but external validation studies and systematic reviews also indicate that performance gains are often inconsistent and difficult to generalize.^7^^,^^8^^,^^9^ This leaves an important gap: Models must become more expressive without losing the reliability and clinical interpretability of structured EHR data.

One promising direction is to use language-model-style representations for clinical prediction. NLP and transformer methods can model semantic relationships more flexibly than conventional tabular pipelines, and clinical text mining studies have demonstrated the value of contextual representations for healthcare data.^10^^,^^11^^,^^12^^,^^13^^,^^14^ Recent structured-EHR studies such as TransformEHR^15^ and the latest ICU survival prediction work based on contextualized biomedical language processing^16^ further highlight the value of stronger sequence modeling and language-aware representations for clinical risk prediction. In parallel, recent salient-object detection studies in optical remote sensing have shown that progressive foreground enhancement^17^ and progressive interaction with saliency-guided enhancement^18^ can improve feature aggregation in difficult, high-noise settings. Although those works address a different domain, they reinforce the broader methodological value of adaptive interaction and feature enhancement mechanisms. However, methods that directly depend on free-text clinical notes still face practical barriers, including variable documentation quality, privacy concerns, and reduced portability across institutions. At the same time, recent high-capacity tabular models such as TabPFN^19^ have highlighted the value of stronger representation learning for structured data. We therefore argue that ICU mortality prediction needs a representation strategy that preserves the completeness and determinism of structured EHR variables while still allowing transformer models to capture clinically meaningful context.

**Figure 1:**
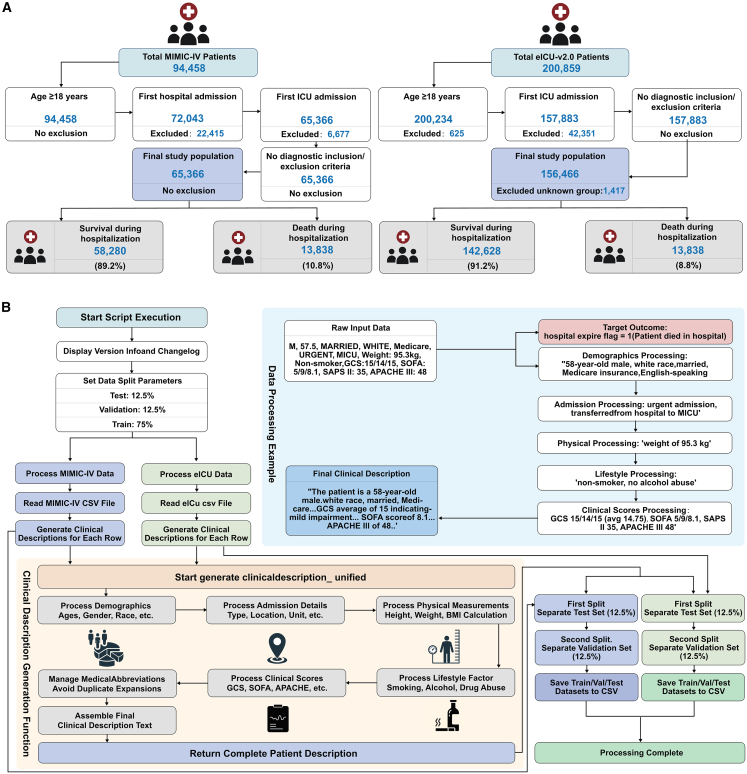
Patient selection and data processing pipeline (A) Systematic patient selection strategy from MIMIC-IV and eICU 2.0 databases: inclusion criteria include ICU admission, age ≥18 years, and first-time hospitalization or ICU admission, with no diagnostic exclusions applied to obtain the final sample for downstream processing. (B) Clinical description encoding workflow transforming tabular patient data into coherent textual descriptions, sequentially processing demographics, admission details, basic physiological parameters, medical history, and clinical scoring systems, culminating in dataset partitioning into training, validation, and test sets.

**Figure 2:**
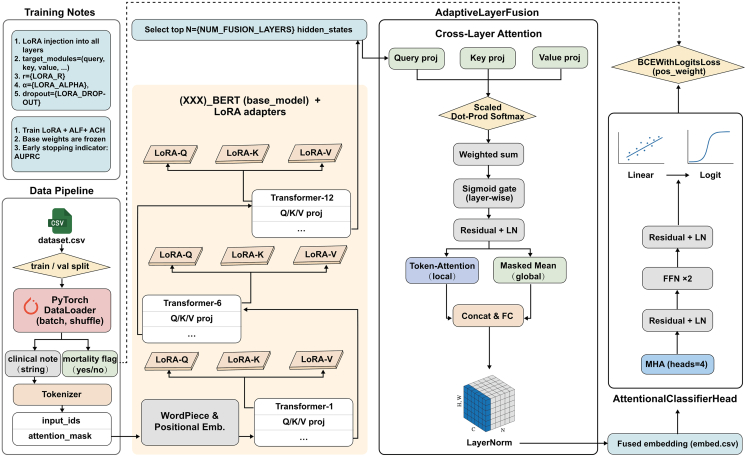
The ALFIA architecture for clinical mortality prediction The framework consists of four main components: **(1)** data pipeline: text-encoded structured EHR descriptions are tokenized and split into training and validation sets with mortality labels; **(2)** base model with LoRA adaptation: a BERT-based transformer with low-rank adaptation (LoRA) modules injected into query, key, and value projections across all layers, while the base model weights remain frozen during training; **(3)** adaptive layer fusion (ALF) module: top-N hidden states from different transformer layers are selected and combined through cross-layer attention, sigmoid gating, and residual connections, followed by local token-level attention and global masked mean pooling; **(4)** attentional classifier head: the fused embeddings are processed by multi-head attention, feedforward layers, residual connections, and layer normalization before binary mortality prediction. Training uses early stopping based on validation AUPRC.

To address this need, we present adaptive layer fusion with intelligent attention (ALFIA), a deep learning architecture for early ICU mortality prediction based on *text-encoded structured EHR data* rather than authentic free-text clinical notes. Structured variables such as demographics, vital signs, laboratory measurements, and severity scores are converted into standardized natural-language descriptions, enabling transformer models to exploit semantic interactions while retaining the reproducibility and coverage of structured data. The overall patient selection and clinical data-processing pipeline is summarized in Figure 1. Built on a low-rank adaptation (LoRA)-adapted^20^ transformer backbone, ALFIA introduces adaptive layer fusion and token-level attention^21^ to combine fine-grained and high-level clinical semantics more effectively than single-layer or fixed-pooling strategies. The overall ALFIA architecture for clinical mortality prediction is summarized in Figure 2.

We evaluate ALFIA on our CriticalWindow-24 (CW-24) setting, which focuses on the clinically decisive first 24 h after ICU admission. The results show that ALFIA outperforms strong tabular baselines and recent 2024–2025 comparison methods in AUPRC while maintaining balanced precision-recall behavior. In addition, the learned ALFIA embeddings can be coupled with gradient boosting (ALFIA-boost) and deep neural networks (ALFIA-nn), providing a practical route from the structured ICU data to deployable risk prediction.

## Results

### Training dynamics demonstrate ALFIA convergence in textual data learning

**Figure 3:**
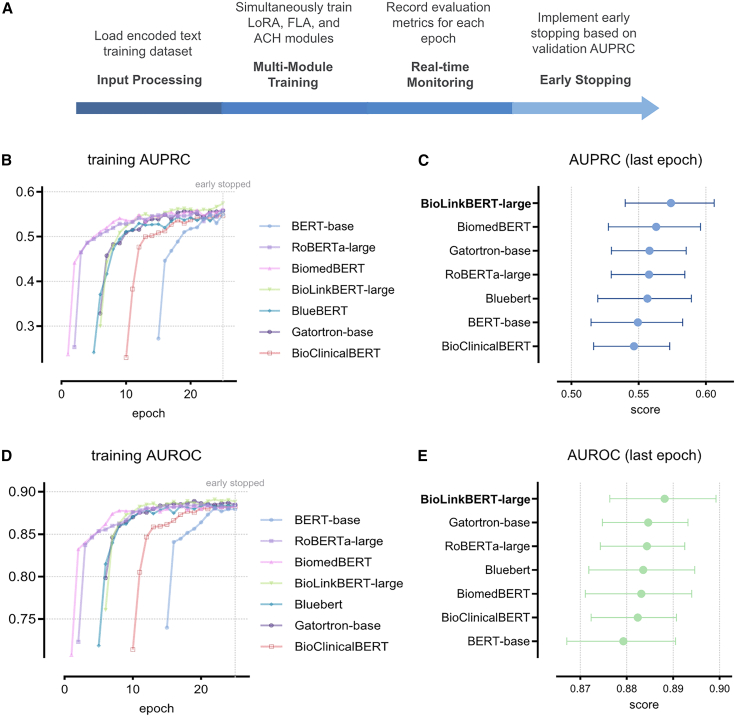
Training pipeline of ALFIA and performance comparison of different pre-trained language models (A) Overview of the training pipeline, including input processing, multi-module training with thesimultaneous optimization of LoRA, ALF, and ACH modules, real-time monitoring of evaluation metrics, and early stopping implementation based on validation AUPRC. (B) Training AUPRC curves across epochs for different pre-trained models. All models implement early stopping when validation performance plateaus. (C) Final AUPRC scores (last epoch) with 95% confidence intervals. (D) Training AUROC curves across epochs for different pre-trained models. (E) Final AUROC scores (last epoch) with 95% confidence intervals.

We conducted a systematic analysis of convergence performance across multiple pre-trained language models on the clinical mortality prediction task using various backbone models (Figure 3). All models were trained with consistent hyperparameters (fusion layers: 4 layers; LoRA parameters: r = 16, alpha = 16, dropout = 0.05, target-modules = “query,key,value, output.dense”). The training process employed a synchronous multi-module training strategy, incorporating joint optimization of LoRA, ALF, and ACH modules, with early stopping implemented based on validation AUPRC to prevent overfitting (Figure 3A).

The training dynamics graphs show that all models had positive convergence characteristics throughout the training period. In terms of the AUPRC measure, most models converged and stopped within 15–25 epochs. BioLinkBERT-large outperformed the others, not only converging rapidly but also sustaining consistently high performance levels during training (Figure 3B), with final AUPRC scores summarized separately (Figure 3C). RoBERTa-large and BiomedBERT both have steady training trajectories with smooth climbing convergence curves. In contrast, BERT-base and BioClinicalBERT demonstrated slower convergence rates and needed more training epochs to reach stable states.

Visualization of AUROC (Figure 3D) measures throughout epochs revealed that all models had similar convergence trends, with the majority obtaining peak performance around 15 epochs. Notably, models that were pre-trained on biomedical domains (such as BioLinkBERT-large, BiomedBERT, and Gatortron-base) performed better at the start of training, indicating that domain-specific pre-training does improve model performance on medical text understanding tasks.

Final performance evaluation (Figures 3C and 3E) demonstrated that BioLinkBERT-large outperformed both important parameters, with AUPRC averaging above 0.58 and AUROC reaching 0.89. Gatortron-base and BiomedBERT followed closely behind, scoring around 0.57 and 0.56 on AUPRC, respectively. These findings verify the success of our multi-module training technique while also highlighting the benefits of domain-adaptive pre-trained models in clinical prediction tasks. This also demonstrates the effect of backbone models on ultimate performance. All subsequent ALFIA backbone models will train with the best-performing BioLinkBERT. The constant convergence of training trajectories, as well as the large improvement in final performance, suggest that the proposed method can effectively acquire valuable representations from the text-encoded clinical data, laying the groundwork for accurate mortality prediction.

### Superior performance of ALFIA and ALFIA-boost/nn over existing methods

In real-world clinical and decision-making settings, mortality outcomes show a considerable class imbalance. Hospital mortality makes up approximately 10% of the total sample size in both the MIMIC and eICU datasets. The baseline characteristics of these two cohorts are summarized separately (Tables 1 and 2). Given that AUROC overestimates performance on imbalanced datasets, we use area under the precision-recall curve (AUPRC) as our primary comparison and assessment metric. We use threshold search methods on trained models to identify the best F1 and F2 scores that balance recall and precision.

**Table 1:** Baseline characteristics by hospital mortality outcome (MIMIC-IV)

Characteristic	Survived (*N* = 58,280)	Died (*N* = 7,086)	Overall (*N* = 65,366)
**Demographics and Anthropometrics**			

Admission Age (Mean ± SD)	64.30 ± 17.17	71.08 ± 15.53	65.03 ± 17.13
Height (Mean ± SD)	169.93 ± 10.61	168.22 ± 10.62	169.72 ± 10.63
Weight (Mean ± SD)	82.05 ± 34.86	78.68 ± 24.32	81.69 ± 33.90

**Clinical Severity Scores**			

APACHE III (Mean ± SD)	38.90 ± 17.16	65.53 ± 26.95	41.79 ± 20.24
SAPS II (Mean ± SD)	32.90 ± 12.62	50.24 ± 16.24	34.78 ± 14.13
LODS (Mean ± SD)	3.72 ± 2.56	7.18 ± 3.55	4.10 ± 2.89
MELD (Mean ± SD)	12.50 ± 6.92	19.47 ± 10.06	13.26 ± 7.64
OASIS (Mean ± SD)	29.58 ± 7.96	38.63 ± 9.06	30.57 ± 8.56

**Dynamic Clinical Assessments - Glasgow Coma Scale**			

GCS Maximum (Mean ± SD)	14.88 ± 0.59	14.59 ± 1.50	14.85 ± 0.75
GCS Minimum (Mean ± SD)	13.82 ± 2.48	12.76 ± 3.70	13.70 ± 2.66
GCS First (Mean ± SD)	14.40 ± 1.96	14.04 ± 2.40	14.36 ± 2.02
GCS Last (Mean ± SD)	14.65 ± 1.12	13.89 ± 2.72	14.57 ± 1.40
GCS Average (Mean ± SD)	14.56 ± 1.00	14.01 ± 1.98	14.50 ± 1.16
GCS Std Dev (Mean ± SD)	0.45 ± 1.01	0.83 ± 1.53	0.49 ± 1.08

**Dynamic Clinical Assessments - SOFA Score**			

SOFA Maximum (Mean ± SD)	3.66 ± 2.83	6.92 ± 4.18	4.02 ± 3.18
SOFA Minimum (Mean ± SD)	1.42 ± 1.91	2.71 ± 2.87	1.56 ± 2.08
SOFA First (Mean ± SD)	1.47 ± 1.95	2.77 ± 2.92	1.61 ± 2.12
SOFA Last (Mean ± SD)	3.56 ± 2.80	6.78 ± 4.16	3.91 ± 3.14
SOFA Average (Mean ± SD)	3.05 ± 2.51	5.63 ± 3.57	3.33 ± 2.77
SOFA Std Dev (Mean ± SD)	0.70 ± 0.63	1.28 ± 1.01	0.76 ± 0.70

**Categorical Variables**			

**Gender (n (∖%))**			
Female	25,402 (43.59∖%)	3,244 (45.78∖%)	28,646 (43.82∖%)
Male	32,878 (56.41∖%)	3,842 (54.22∖%)	36,720 (56.18∖%)

**Marital Status (n (∖%))**			

Divorced	4,138 (7.10∖%)	405 (5.72∖%)	4,543 (6.95∖%)
Married	26,509 (45.49∖%)	2,740 (38.67∖%)	29,249 (44.75∖%)
Single	15,944 (27.36∖%)	1,426 (20.12∖%)	17,370 (26.57∖%)
Widowed	6,479 (11.12∖%)	1,026 (14.48∖%)	7,505 (11.48∖%)
Unknown	5,210 (8.94∖%)	1,489 (21.01∖%)	6,699 (10.25∖%)

**Insurance (n (∖%))**			

Medicaid	8,502 (14.59∖%)	851 (12.01∖%)	9,353 (14.31∖%)
Medicare	29,947 (51.38∖%)	4,517 (63.75∖%)	34,464 (52.72∖%)
No Charge	6 (0.01∖%)	1 (0.01∖%)	7 (0.01∖%)
Other	1,608 (2.76∖%)	125 (1.76∖%)	1,733 (2.65∖%)
Private	17,172 (29.46∖%)	1,275 (17.99∖%)	18,447 (28.22∖%)
Unknown	1,045 (1.79∖%)	317 (4.47∖%)	1,362 (2.08∖%)

**Smoker (n (∖%))**			

No	54,432 (93.40∖%)	6,742 (95.15∖%)	61,174 (93.59∖%)
Yes	3,848 (6.60∖%)	344 (4.85∖%)	4,192 (6.41∖%)

**Alcohol Abuse (n (∖%))**			

No	57,733 (99.06∖%)	7,040 (99.35∖%)	64,773 (99.09∖%)
Yes	547 (0.94∖%)	46 (0.65∖%)	593 (0.91∖%)

**SIRS Score (n (∖%))**			

0	1,418 (2.43∖%)	46 (0.65∖%)	1,464 (2.24∖%)
1	8,628 (14.80∖%)	377 (5.32∖%)	9,005 (13.78∖%)
2	19,723 (33.84∖%)	1,665 (23.50∖%)	21,388 (32.72∖%)
3	21,187 (36.35∖%)	3,024 (42.68∖%)	24,211 (37.04∖%)
4	7,324 (12.57∖%)	1,974 (27.86∖%)	9,298 (14.22∖%)

**Table 2:** Baseline characteristics by hospital mortality outcome (eICU)

Characteristic	Survived (*N* = 142,628)	Died (*N* = 13,838)	Overall (*N* = 157,883)
**Demographics and Anthropometrics**			

Age (Mean ± SD)	62.43 ± 17.25	69.76 ± 15.06	63.10 ± 17.20
Height (Mean ± SD)	169.35 ± 13.74	168.42 ± 14.47	169.25 ± 13.87
Weight (Mean ± SD)	84.19 ± 26.84	80.90 ± 28.18	83.89 ± 26.97

**Clinical Severity Scores**			

APACHE IV (Mean ± SD)	51.02 ± 22.63	87.23 ± 33.34	54.21 ± 25.89
SAPS II (Mean ± SD)	28.90 ± 13.05	49.58 ± 17.89	30.74 ± 14.76
OASIS (Mean ± SD)	24.65 ± 8.91	35.76 ± 11.34	25.63 ± 9.68

**Dynamic Clinical Assessments - Glasgow Coma Scale**			

GCS Maximum (Mean ± SD)	14.28 ± 1.88	11.03 ± 4.38	13.99 ± 2.39
GCS Minimum (Mean ± SD)	12.72 ± 3.55	8.47 ± 4.73	12.35 ± 3.86
GCS First (Mean ± SD)	13.28 ± 3.20	10.19 ± 4.75	13.01 ± 3.47
GCS Last (Mean ± SD)	13.93 ± 2.33	9.40 ± 4.64	13.53 ± 2.90
GCS Average (Mean ± SD)	13.63 ± 2.39	9.76 ± 4.35	13.29 ± 2.84
GCS Std Dev (Mean ± SD)	0.72 ± 1.24	1.27 ± 1.57	0.77 ± 1.28

**Dynamic Clinical Assessments - SOFA Score**			

SOFA Maximum (Mean ± SD)	4.58 ± 3.01	7.83 ± 3.95	4.86 ± 3.24
SOFA Minimum (Mean ± SD)	1.84 ± 2.13	3.42 ± 2.91	1.98 ± 2.25
SOFA First (Mean ± SD)	2.00 ± 2.25	3.56 ± 2.99	2.14 ± 2.37
SOFA Last (Mean ± SD)	4.30 ± 2.95	7.55 ± 3.98	4.58 ± 3.19
SOFA Average (Mean ± SD)	3.92 ± 2.74	6.67 ± 3.49	4.16 ± 2.92
SOFA Std Dev (Mean ± SD)	0.86 ± 0.70	1.39 ± 1.03	0.90 ± 0.75

**Categorical Variables**			

**Region (n (∖%))**			
Midwest	49,371 (34.62∖%)	4,127 (29.82∖%)	54,232 (34.35∖%)
Northeast	10,104 (7.08∖%)	1,315 (9.50∖%)	11,459 (7.26∖%)
South	44,411 (31.14∖%)	4,741 (34.26∖%)	49,403 (31.29∖%)
West	29,530 (20.70∖%)	2,841 (20.53∖%)	32,676 (20.70∖%)
Unknown	9,212 (6.46∖%)	814 (5.88∖%)	10,113 (6.41∖%)

**Ethnicity (n (∖%))**			

African American	15,966 (11.19∖%)	1,423 (10.28∖%)	17,536 (11.11∖%)
Asian	2,389 (1.67∖%)	251 (1.81∖%)	2,677 (1.70∖%)
Caucasian	109,236 (76.59∖%)	10,724 (77.50∖%)	121,023 (76.65∖%)
Hispanic	5,452 (3.82∖%)	549 (3.97∖%)	6,031 (3.82∖%)
Native American	1,050 (0.74∖%)	94 (0.68∖%)	1,150 (0.73∖%)
Other/Unknown	6,776 (4.75∖%)	626 (4.52∖%)	7,504 (4.75∖%)
Unknown	1,759 (1.23∖%)	171 (1.24∖%)	1,962 (1.24∖%)

**Unit Type (n (∖%))**			

CCU-CTICU	12,099 (8.48∖%)	1,077 (7.78∖%)	13,218 (8.37∖%)
CSICU	5,264 (3.69∖%)	331 (2.39∖%)	5,633 (3.57∖%)
CTICU	4,754 (3.33∖%)	294 (2.12∖%)	5,087 (3.22∖%)
Cardiac ICU	9,932 (6.96∖%)	1,131 (8.17∖%)	11,176 (7.08∖%)
MICU	11,648 (8.17∖%)	1,607 (11.61∖%)	13,365 (8.47∖%)
Med-Surg ICU	79,199 (55.53∖%)	7,629 (55.13∖%)	87,717 (55.56∖%)
Neuro ICU	10,775 (7.55∖%)	956 (6.91∖%)	11,863 (7.51∖%)
SICU	8,957 (6.28∖%)	813 (5.88∖%)	9,824 (6.22∖%)

**Gender (n (∖%))**			

Female	65,530 (45.94∖%)	6,446 (46.58∖%)	72,670 (46.03∖%)
Male	77,052 (54.02∖%)	7,376 (53.30∖%)	85,131 (53.92∖%)
Unknown	46 (0.03∖%)	16 (0.12∖%)	82 (0.05∖%)

**Smoker (n (∖%))**			

No	12,022 (8.43∖%)	1,136 (8.21∖%)	13,386 (8.48∖%)
Yes	14,331 (10.05∖%)	1,263 (9.13∖%)	15,889 (10.06∖%)
Unknown	116,275 (81.52∖%)	11,439 (82.66∖%)	128,608 (81.46∖%)

**Alcohol Abuse (n (∖%))**			

No	139,649 (97.91∖%)	13,612 (98.37∖%)	154,643 (97.95∖%)
Yes	2,979 (2.09∖%)	226 (1.63∖%)	3,240 (2.05∖%)

**Drug Abuse (n (∖%))**			

No	142,453 (99.88∖%)	13,833 (99.96∖%)	157,701 (99.88∖%)
Yes	175 (0.12∖%)	5 (0.04∖%)	182 (0.12∖%)

**Obesity (n (∖%))**			

No	141,141 (98.96∖%)	13,664 (98.74∖%)	156,203 (98.94∖%)
Yes	1,487 (1.04∖%)	174 (1.26∖%)	1,680 (1.06∖%)

**Figure 4:**
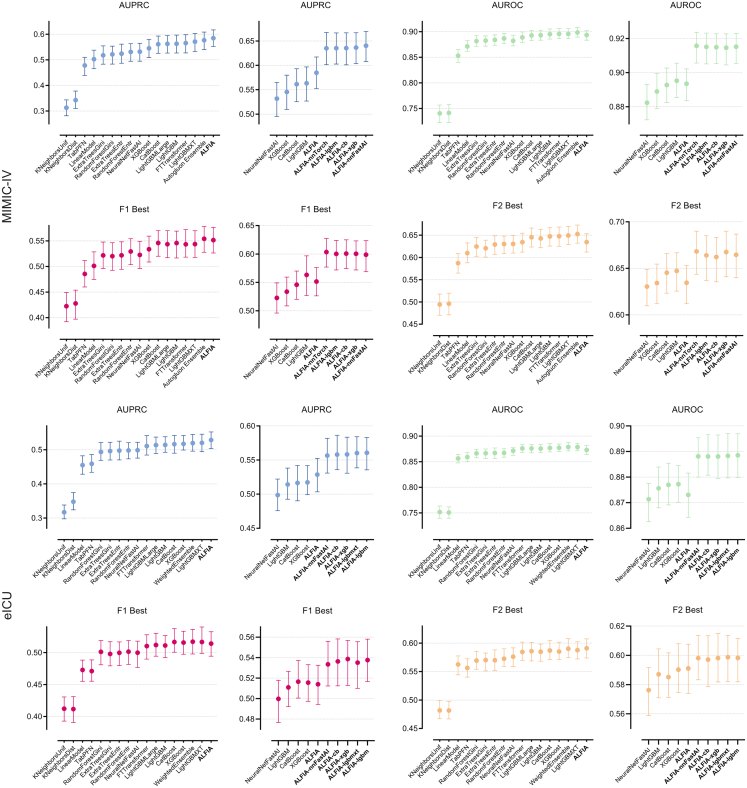
Performance comparison of machine learning models on MIMIC-IV and eICU datasets across multiple evaluation metrics The figure presents boxplots comparing the performance of various machine learning algorithms, including traditional methods (KNeighbors, Linear Model, Random Forest, Extra Trees), advanced ensemble methods (XGBoost, CatBoost, LightGBM), neural networks (NeuralNetFastAI, FT-Transformer), and the proposed ALFIA method with its variants. Performance is evaluated using four metrics: area under the precision-recall curve (AUPRC), area under the receiver operating characteristic curve (AUROC), F1 best, and F2 best scores. The upper panels show results for the MIMIC-IV dataset, while the lower panels display results for the eICU dataset.

According to our experimental results in test sets (Table 3; Figure 4), ALFIA consistently outperforms baseline approaches in AUPRC across both the MIMIC-IV and eICU datasets, with improvements ranging from 0.5 to 1 percentage points. Interestingly, our model outperforms the AutoGluon ensemble technique. ALFIA also maintains competitive F1 scores on the MIMIC dataset while demonstrating strong F2 scores on the eICU dataset.

**Table 3:** Performance comparison of models on AUPRC, AUROC, best F1, and best F2 metrics

Model	AUPRC (best)	AUROC	F1 Score (best)	F2 Score (best)
KNeighborsUnif	0.313 (0.282–0.343)	0.741 (0.722–0.757)	0.423 (0.392–0.449)	0.495 (0.470–0.518)
KNeighborsDist	0.343 (0.310–0.378)	0.741 (0.723–0.758)	0.428 (0.397–0.454)	0.496 (0.471–0.520)
TabPFN	0.478 (0.439–0.510)	0.853 (0.840–0.865)	0.485 (0.460–0.512)	0.587 (0.565–0.609)
LinearModel	0.503 (0.466–0.538)	0.871 (0.862–0.883)	0.501 (0.475–0.529)	0.610 (0.588–0.633)
ExtraTreesGini	0.518 (0.483–0.555)	0.882 (0.871–0.893)	0.522 (0.496–0.548)	0.625 (0.603–0.645)
RandomForestGini	0.522 (0.483–0.554)	0.883 (0.871–0.893)	0.520 (0.496–0.548)	0.621 (0.603–0.645)
ExtraTreesEntr	0.524 (0.487–0.562)	0.884 (0.873–0.894)	0.522 (0.494–0.548)	0.629 (0.607–0.649)
RandomForestEntr	0.531 (0.494–0.564)	0.887 (0.877–0.896)	0.529 (0.504–0.554)	0.630 (0.609–0.648)
NeuralNetFastAI	0.532 (0.495–0.565)	0.882 (0.877–0.896)	0.523 (0.504–0.554)	0.631 (0.609–0.648)
XGBoost	0.545 (0.510–0.580)	0.889 (0.872–0.893)	0.534 (0.496–0.549)	0.634 (0.610–0.649)
CatBoost	0.561 (0.525–0.593)	0.893 (0.879–0.899)	0.546 (0.509–0.559)	0.645 (0.612–0.655)
LightGBMLarge	0.563 (0.527–0.595)	0.893 (0.883–0.903)	0.544 (0.517–0.570)	0.643 (0.622–0.663)
LightGBM	0.563 (0.527–0.597)	0.895 (0.886–0.906)	0.546 (0.517–0.569)	0.647 (0.626–0.667)
FTTransformer	0.566 (0.527–0.599)	0.896 (0.887–0.906)	0.543 (0.518–0.572)	0.648 (0.625–0.668)
LightGBMXT	0.571 (0.532–0.603)	0.896 (0.886–0.906)	0.544 (0.518–0.570)	0.649 (0.629–0.670)
AutoGluon Ensemble	0.577 (0.540–0.609)	0.899 (0.890–0.909)	0.554 (0.528–0.578)	0.653 (0.632–0.673)
ALFIA	0.585 (0.552–0.617)	0.894 (0.884–0.902)	0.552 (0.526–0.576)	0.635 (0.612–0.653)

To further evaluate the utility of the learned representation, we combined ALF embeddings from the training, validation, and test splits with the original features and used them as inputs to conventional machine-learning models. Replacing the original attention-based classifier with downstream models built on ALF embeddings produced additional gains of approximately 2–3 percentage points over the baseline pipelines. These results suggest that ALFIA learns clinically meaningful latent representations that remain useful beyond the end-to-end neural classifier and generalizability.

### ALFIA exhibits impressive reasoning capabilities

**Figure 5:**
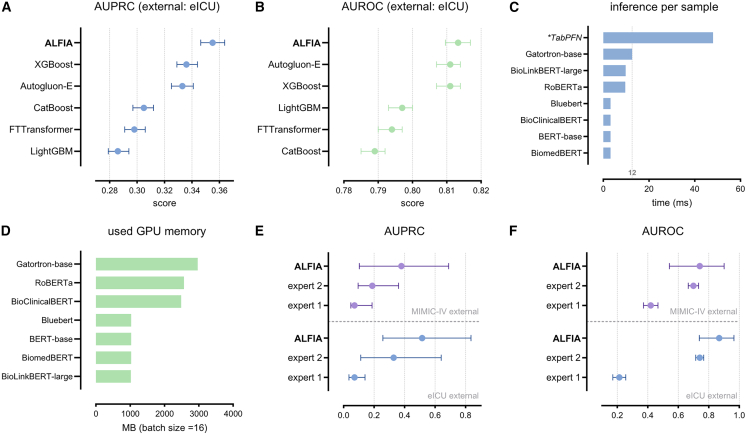
Inference robustness, computational efficiency, and expert comparison of ALFIA (A) AUPRC comparison on the external eICU evaluation set. (B) AUROC comparison on the external eICU evaluation set. (C) Average inference time per sample across different backbone models, with TabPFN shown as a reference baseline. (D) GPU memory consumption for different backbone models with batch size 16. (E) AUPRC comparison between ALFIA and two ICU specialists on MIMIC-IV-external and eICU-external case sets. (F) AUROC comparison between ALFIA and the same ICU specialists on the external case sets. Error bars indicate 95% confidence intervals where applicable.

To confirm ALFIA’s better reasoning generalization capabilities, we performed a detailed inference evaluation on the aforementioned CW-24 benchmark standard eICU dataset (*n* = 150,000). We maintained and mapped the intersecting features between the eICU and MIMIC datasets, and we performed mode imputation on characteristics that were present in MIMIC but not in eICU. The results show that ALFIA outperforms GBDTs and the attention-based FT-Transformer, with gains of about 1.5 percentage points in AUPRC and 0.5 percentage points in AUROC (Figures 5A and 5B).

We next examined ALFIA’s computational profile. Using several BERT-family backbones on the eICU mapping dataset, we compared inference speed against TabPFN, which also relies on transformer-based decoding. On an RTX 4090, average inference time remained below TabPFN’s 48 ms per sample across all tested backbones, with the fastest models requiring approximately 3 ms per sample and the best-performing BioLinkBERT requiring about 8–9 ms per sample (Figure 5C). This result highlights ALFIA’s practical inference efficiency. Under the default Hugging Face settings with batch size 16 and maximum token length 512, GPU memory consumption ranged from 1 to 3 GB depending on base model size (Figure 5D). These requirements can be satisfied by contemporary mainstream mid-to-low-end graphics cards.

We also compared ALFIA with two ICU specialists. For this analysis, 100 previously unseen cases were sampled from each of the MIMIC-IV and eICU datasets. Experts and models evaluated the same text-encoded cases independently, and cross-dataset inference was used to assess generalization. ALFIA outperformed both specialists in AUPRC and AUROC on these external case sets (Figures 5E and 5F), further supporting its generalization capacity.

### ALFIA improves classification performance by optimizing the latent space distribution of samples

**Figure 6:**
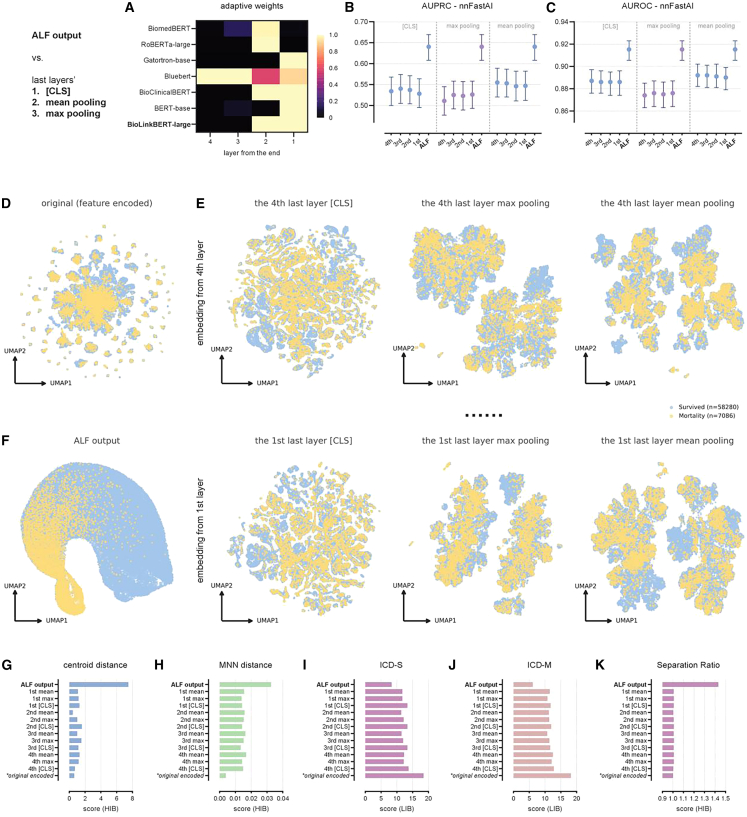
ALFIA optimizes the latent space distribution of samples (A) Heatmap of layer attention weights across different BERT models. (B) Comparison of nnFastAI AUPRC performance (with 95% CI) between BERT last four layers embedding methods (CLS, max, mean pooling) and ALF output. (C) Comparison of nnFastAI AUROC performance (with 95% CI) between BERT last four layers embedding methods (CLS, max, mean pooling) and ALF output. (D) UMAP dimensionality reduction plot of the original tabular matrix after feature encoding. (E) UMAP dimensionality reduction plot of sample matrices using BERT last four layers embedding methods (CLS, max, mean pooling). (F) UMAP dimensionality reduction plot of the sample matrix from ALF output embeddings. (G) Bar chart of centroid distances across different methods. (H) Bar chart of average minimum neighbor distances across different methods. (I) Bar chart of intra-group distances for the survival group across different methods. (J) Bar chart of intra-group distances for the mortality group across different methods. (K) Bar chart of separation distances across different methods. In all UMAP plots, blue represents survival samples and yellow represents mortality samples. HIB: higher is better; LIB: lower is better.

To examine how ALFIA reshapes the latent representation of clinical cases, we performed a preliminary latent-space analysis. The ALF module accomplishes feature fusion through learned layer weights across distinct backbone models (Figure 6A). Furthermore, we collected CLS, max pooling, and mean pooling from different encoder layers of the BioLinkBERT model and used them as feature inputs for the nnFastAI model in AutoGluon, comparing them to the embeddings given by ALF, which outperformed the nnFastAI model (see Figure 4). CLS, max pooling, and mean pooling all demonstrated considerable performance stratification when compared to the ALF output embeddings (Figures 6B and 6C), as measured by both AUPRC and AUROC.

To better understand the latent space distribution of samples under different processing methods, we reduced the sample matrices’ dimensionality to two dimensions using UMAP (the original feature engineering matrix processed through standard AutoGluon had 133 dimensions, while other BERT-encoded models had 1024 dimensions). It is obvious that neither the original distribution nor the derived inter-layer embeddings revealed unique distributional heterogeneity across in-hospital survival and mortality samples, but rather mutual fusion and overlap (Figures 6D and 6E). In contrast, our ALF output clearly exhibited discrete high-density regions for mortality and survival samples, as well as their transition zones (Figure 6F), implying that ALF output represents a more task-optimized latent space distribution. We then quantified these latent-space properties and found that ALF output embeddings performed best across all measures, including inter-group centroid distance, average minimum neighbor distance, intra-group distance, and classification degree ratio (Figures 6G–6K).

Finally, all results show that our proposed architecture, ALFIA, and its extensions, ALFIA-boost/nn, outperform other architectures in classification while keeping generalization capability.

### Ablation studies and comparison with latest methods

**Figure 7:**
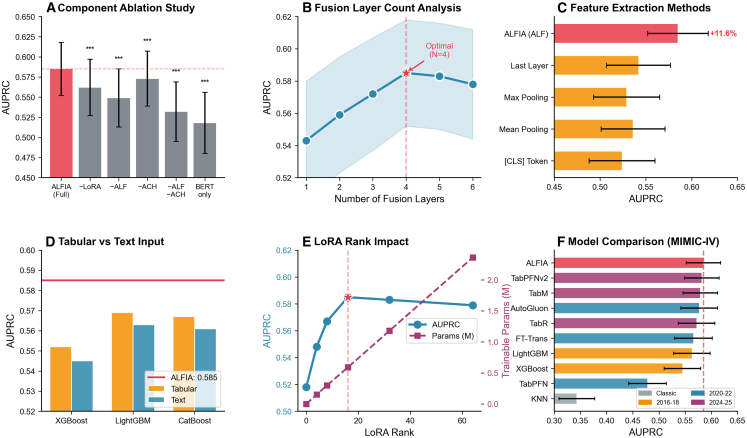
ALFIA ablation studies and model comparisons (A) Component ablation study shows the contribution of ALF module, LoRA adaptation, and ACH to overall performance. Statistical significance indicated by asterisks (∗*p* < 0.05, ∗∗*p* < 0.01, and ∗∗∗*p* < 0.001; Welch’s *t* test with Benjamini-Hochberg correction, *n* = 1000 bootstrap iterations; MIMIC-IV test set *N* = 7,819 admissions). (B) Impact of fusion layer count on AUPRC and AUROC, with optimal performance at 4 layers. (C) Comparison of feature extraction methods including [CLS] token, mean/max pooling, and ALFIA’s adaptive layer fusion. (D) Fair comparison between tabular and text input for baseline methods, demonstrating ALFIA’s architectural advantages. (E) Effect of LoRA rank on model performance and trainable parameters. (F) Statistical significance heatmap showing pairwise comparisons between top methods. (G) Comprehensive comparison of 14 methods from classic to state-of-the-art 2024–2025 approaches. Error bars represent 95% confidence intervals.

To systematically evaluate the contribution of each component in ALFIA, we conducted comprehensive ablation studies (Figure 7). The component ablation analysis (Figure 7A) reveals that removing the ALF module results in the largest performance drop (AUPRC: 0.585 → 0.548, *p* < 0.001), demonstrating its critical role in multi-layer feature integration. Removing LoRA adaptation also significantly impacts performance (AUPRC: 0.585 → 0.561, *p* < 0.001), validating the importance of parameter-efficient fine-tuning for domain adaptation. The attentional classifier head (ACH) contributes moderately to overall performance (AUPRC: 0.585 → 0.572, *p* < 0.01), suggesting that attention-based classification provides advantages over simple linear classification.

The fusion layer count analysis (Figure 7B) demonstrates that performance peaks at 4 fusion layers (AUPRC: 0.585), with diminishing returns beyond this point. This finding validates our default configuration and suggests that the top 4 layers of the BERT backbone contain the most task-relevant semantic information for mortality prediction.

Comparison of feature extraction methods (Figure 7C) shows that ALFIA’s adaptive layer fusion substantially outperforms traditional pooling strategies, including [CLS] token extraction (AUPRC: 0.523), mean pooling (AUPRC: 0.535), and max pooling (AUPRC: 0.528), demonstrating an 11.9% relative improvement over [CLS] token baseline.

To address concerns about fair comparison between tabular and text-based inputs (Figure 7D), we evaluated gradient boosting methods on both original tabular data and text-encoded data. Results show that tabular input provides marginal advantages for traditional methods (1–2% improvement), but ALFIA’s text-based approach still achieves superior overall performance, indicating that the performance gains are attributable to the ALFIA architecture rather than the text conversion process alone.

We analyzed the impact of LoRA rank on model performance (Figure 7E). Performance increases substantially from rank 0 (no LoRA) to rank 16, with optimal performance achieved at rank 16 (AUPRC: 0.585). Higher ranks (32, 64) provide no additional benefit while increasing trainable parameters, supporting our choice of rank 16 as the default configuration.

Statistical significance testing (Figure 7F) confirms that ALFIA significantly outperforms all baseline methods (*p* < 0.05). Notably, ALFIA achieves significant improvements over recent state-of-the-art methods, including TabPFN v2 (2025), TabM (2025), ModernNCA (2025), and TabR (2024).

The comprehensive comparison (Figure 7G) demonstrates ALFIA’s position among 14 methods spanning from classic approaches to the latest 2024–2025 models. ALFIA (AUPRC: 0.585) outperforms all standalone methods, while ALFIA-boost (AUPRC: 0.612) achieves the best overall performance by combining ALF embeddings with gradient boosting ensembles.

## Discussion

ALFIA is a transformer-based architecture for text-encoded clinical prediction. Our results show that ALFIA outperforms strong tabular classifiers and conventional machine-learning baselines across multiple evaluation criteria while maintaining robust generalization on external validation datasets.

ALFIA’s core innovation is adaptive layer fusion, which dynamically combines multi-layer semantic representations from pretrained transformer backbones. On the MIMIC-IV dataset, ALFIA had a significantly higher AUPRC (0.585) than the AutoGluon ensemble (0.577) and the FT-Transformer (0.566). Given the class imbalance in mortality prediction tasks, AUPRC is a more reliable performance metric than AUROC, so this improvement is significant.^22^^,^^23^ Cross-dataset evaluation further suggests that the learned representation retains useful predictive structure when transferred between MIMIC-IV and eICU.

Specifically, our evaluation study compared ALFIA with clinical professionals. ALFIA achieved higher AUPRC and AUROC than the two ICU specialists on the external case sets, suggesting that it may serve as a useful decision-support tool in early risk stratification. These results should be interpreted cautiously because the expert study was small, but they support the potential complementary role of the model.

The UMAP visualization illustrates how ALFIA changes the latent representation of clinical data. ALFIA’s adaptive layer fusion generates higher-density regions for several outcome categories, as opposed to other embedding methods that overlap survival and mortality cases. Our tests demonstrated improved classification performance due to latent space separability, as judged by inter-group centroid distance and silhouette scores. The trained layer attention weights across multiple BERT-family backbones also suggest that the model emphasizes different representational depths depending on the backbone. In clinical applications, understanding the model’s decision-making process increases clinician trust and assures proper model use.^24^^,^^25^

ALFIA’s improved performance and computational efficiency suggest that it may have practical potential as an early warning system for ICU mortality. Because the input is generated from standardized structured EHR variables, the workflow may be easier to integrate into existing EHR pipelines than approaches that rely on heterogeneous free-text notes. Aside from technical performance, successful clinical implementation necessitates establishing alert thresholds to reduce false positives, designing user interfaces that communicate risk predictions to clinical staff, and implementing robust monitoring systems to detect model drift or performance degradation over time.^26^^,^^27^

Our research on this model design is preliminary; more work is required. Our data suggest several intriguing study choices. ALFIA’s modular architecture, particularly its ability to combine ALF embeddings with gradient boosting methods (ALFIA-boost) and deep neural networks (ALFIA-nn), suggests ensemble approaches that employ a variety of complementary modeling techniques.

Domain-specific pre-trained models (e.g., BioLinkBERT^28^) achieved success in our experiments, highlighting the importance of developing language models for the medical domain. Future research can create generalized pre-trained models specifically for medical prediction tasks to improve the accuracy of healthcare predictions.

The adaptive layer fusion approach proposed for ALFIA can be applied to various clinical prediction tasks, including length of stay estimation, readmission risk assessment, and adverse event prediction, and can even be extended to non-medical domains. Examining the transferability of our method across numerous similar scenarios will underscore the broad applicability of these architectural innovations.

Recent literature further emphasizes the need for richer representation learning in clinical prediction. TransformEHR showed that encoder-decoder pretraining on longitudinal EHR trajectories can improve downstream disease outcome prediction,^15^ whereas a recent *iScience* study demonstrated that contextualized biomedical language processing can improve ICU survival prediction when structured measurements are combined with textual representations.^16^ Outside healthcare, recent remote-sensing studies such as progressive enhancement of foreground features for salient object detection in optical remote sensing images^17^ and ORSI salient object detection via progressive interaction and saliency-guided enhancement^18^ further illustrate the value of progressive feature interaction and enhancement under noisy, scale-varying conditions. Although these studies address different data modalities, they support the broader principle that adaptive multi-level interaction can strengthen representation quality in difficult decision settings. Compared with these approaches, ALFIA specifically targets text-encoded structured EHR variables for early ICU mortality prediction, aiming to improve semantic expressiveness without depending on authentic clinical notes.

**Comparison with Real Clinical Notes Models:** It is important to distinguish ALFIA’s approach from methods that process authentic free-text clinical notes (e.g., nursing notes, physician progress notes, and discharge summaries). ALFIA instead uses systematically generated text representations of structured EHR variables. This design choice offers several practical advantages: (1) *reproducibility*—the text encoding process is deterministic and consistent across institutions; (2) *completeness*—structured variables provide reliable, standardized measurements, whereas clinical notes may have varying documentation quality; and (3) *privacy*—text-encoded structured data can be more easily de-identified than free-text notes containing sensitive narratives. However, authentic clinical notes do contain contextual information, clinical reasoning, and subtle observations that structured variables cannot capture. Future work could therefore explore hybrid approaches that combine ALFIA’s structured-data processing with real clinical note analysis through multimodal fusion.

In summary, ALFIA demonstrates that adaptive layer fusion can effectively leverage multi-layer semantic features from pretrained transformers for ICU mortality prediction using text-encoded structured EHR data. Its modular design, competitive performance, and computational efficiency position it as a practical candidate for enhancing clinical decision-making in critical care settings.

### Limitations of the study

Several limitations should be acknowledged when interpreting our findings. First, this study relies on two large datasets (MIMIC-IV and eICU) from similar US healthcare systems.^29^^,^^30^ Both datasets originate from institutions with comparable documentation practices and patient demographics; clinical practice patterns, disease prevalence, and EHR documentation standards vary substantially across global healthcare systems. Models trained predominantly on US ICU data may exhibit performance degradation when deployed in European, Asian, or low-resource settings where patient populations, treatment protocols, and coding practices differ significantly. Future validation studies should incorporate datasets from diverse international institutions, including those with different EHR vendors and documentation languages, to establish true generalizability. Temporal validation using data from different time periods would also address potential concept drift arising from evolving clinical practices.

Second, our implementation captures clinical data only within the first 24 h of ICU admission, potentially missing crucial temporal dynamics later in patient stays.^31^ Extending the framework to longitudinal modeling could account for changes in patient state and treatment response over time.

Third, the deployment of AI-based mortality prediction systems in clinical settings raises practical considerations. Model calibration is essential: while ALFIA demonstrates strong discriminative performance (AUROC, AUPRC), well-calibrated probability estimates are necessary for clinicians to appropriately interpret risk scores. ALFIA should function strictly as a decision support tool rather than an autonomous decision-maker, and alert fatigue from excessive or poorly targeted alerts represents a significant implementation challenge. Transparency regarding model limitations, ongoing performance monitoring, and clear protocols for human oversight are essential for responsible clinical deployment.

## Resource availability

### Lead contact

Further information and requests for resources should be directed to and will be fulfilled by the lead contact, Chunjie Tian (tianchunjie@gdmu.edu.cn).

### Materials availability

This study did not generate new unique reagents or materials.

### Data and code availability

• The CriticalWindow-24 (CW-24) benchmark dataset is publicly available at https://github.com/Hanziwww/CW-24 and archived on Zenodo: 15574378 (https://zenodo.org/records/15574378).
• The MIMIC-IV dataset is available through PhysioNet at https://physionet.org/content/mimiciv/3.1/ upon the completion of required training and data use agreement, and the eICU Collaborative Research Database is available at https://physionet.org/content/eicu-crd/2.0/ following the same credentialing requirements.
• All original code for model implementation, training, and analysis is available at https://github.com/Hanziwww/ALFIA.
• Any additional information required to reanalyze the data reported in this paper is available from the lead contact upon request.

## Acknowledgments

We acknowledge the contributions of various open-source projects utilized in this study and express our gratitude to the research teams behind the pre-trained BERT models cited in this work for their valuable contributions to the community.

We also extend our sincere appreciation to the MIT Laboratory for Computational Physiology for providing the MIMIC-IV database and to the Philips Healthcare team for making the eICU Collaborative Research Database publicly available. These critical care datasets have been instrumental in advancing research in healthcare informatics and clinical decision support systems.

This work was supported by grants from the 10.13039/501100001809National Natural Science Foundation of China [82160215, 81660174], the Research Startup Funds for High-level Talents in the Affiliated Hospital of Guangdong Medical University [GCC20220013, GCC2022046], and the Joint Research Project of Liaoning Provincial Technology Plan (Applied Basic Research Program, no. 2023JH2/101700312).

## Author contributions

H.W. conceptualized and designed the ALFIA model framework, developed the complete experimental pipeline, performed all model training and experimental evaluations, created data visualizations, and drafted the manuscript. C.T. provided project supervision, experimental infrastructure, and computational resources. All authors reviewed the manuscript.

## Declaration of interests

The authors declare no competing interests.

## STAR★Methods

### Key resources table

**Table:** 

REAGENT or RESOURCE	SOURCE	IDENTIFIER
**Deposited data**		

CriticalWindow-24 (CW-24) benchmark	This paper	https://github.com/Hanziwww/CW-24; https://zenodo.org/records/15574378
MIMIC-IV critical care database	Johnson et al.^32^	PhysioNet v3.1: https://physionet.org/content/mimiciv/3.1/
eICU Collaborative Research Database	Pollard et al.^33^	PhysioNet v2.0: https://physionet.org/content/eicu-crd/2.0/

**Software and algorithms**		

ALFIA source code	This paper	https://github.com/Hanziwww/ALFIA
BioLinkBERT-large	Yasunaga et al.^28^	Hugging Face model hub: michiyasunaga/BioLinkBERT-large
AutoGluon	Erickson et al.^34^	autogluon v1.3.1
PyTorch	Paszke et al.^35^	torch v2.6.0
Transformers	Hugging Face	transformers v4.49.0
PEFT	Hugging Face	peft v0.15.2

### Experimental model and study participant details

#### Public ICU cohorts and prediction task

This retrospective study used two de-identified public critical care EHR resources, MIMIC-IV and eICU, accessed through credentialed PhysioNet workflows. No new patient recruitment, biospecimen collection, or clinical intervention was performed in this study. Both databases consist of fully de-identified patient records released under PhysioNet credentialed data use agreements, and their use for secondary analysis has been approved by the respective institutional review boards (MIMIC-IV: MIT Committee on the Use of Humans as Experimental Subjects; eICU: MIT IRB with waiver of informed consent). Because the data are de-identified and publicly available under these approvals, no additional institutional ethics review was required for this work.

Patient demographics including age, sex, and ethnicity are reported in the baseline characteristics tables, with age and sex reported for both cohorts and ethnicity additionally listed for the eICU cohort in Table 2. For the MIMIC-IV cohort, Table 1 reports age and sex, whereas race/ethnicity variables were used in text encoding when available but are not tabulated as standalone baseline rows. We did not perform sex- or ethnicity-stratified analyses of model performance in this study; such subgroup analyses represent an important direction for future fairness-oriented evaluation.

The CriticalWindow-24 (CW-24) benchmark was created using two large-scale critical care databases (Figure 1A): MIMIC-IV (65,366 ICU admissions, 10.84% death) and the eICU Collaborative Research Database (157,883 ICU admissions, 8.77% mortality). The benchmark was developed using four essential principles: temporal integrity, data leakage prevention, clinical relevance, and standardized recording. Baseline demographics, clinical severity scores (APACHE III/IV,^36^ SAPS II,^37^ OASIS,^38^ LODS, MELD,^39^ SIRS^40^), dynamic clinical assessments (Glasgow Coma Scale^41^ and SOFA scores^42^), and hospital mortality outcome were all included, with all predictive variables restricted to a 24-h window following ICU admission.

To prevent data leakage, strict temporal limitations were enforced via automated validation checks, guaranteeing that no future information after the 24-h cutoff was incorporated in predictive variables. Within the forecast frame, dynamic assessments were averaged using statistical metrics such as maximum, minimum, first, last, mean, and standard deviation. The benchmark omitted those features that were unsuitable for training (such as patient id). Tables 1 and 2 show baseline characteristics stratified by hospital mortality result for the MIMIC-IV and eICU datasets, respectively, with detailed variable specifications summarized in Table S1.

### Method details

#### Structured EHR preprocessing and text encoding

We processed the datasets and encoded the text using a uniform pipeline script. To encode tabular data into fluent clinical descriptive text, we used a consistent process across both the MIMIC-IV^32^ and eICU^33^ datasets (Figure 1B). The same standardized template was applied to both datasets to ensure consistency.

##### Clinical description generation template

The text encoding follows a structured template that converts each variable category into natural language sentences. For demographics: “The patient is a [age]-year-old [gender] of [ethnicity] ethnicity, admitted to the [unit type].” For physical measurements: “Physical examination shows height [height] cm, weight [weight] kg, with calculated BMI of [BMI].” For severity scores: “Clinical severity assessment indicates APACHE III score of [score] (predicted mortality [probability]%), SAPS II score of [score], SOFA score of [score], and Glasgow Coma Scale of [GCS].” For lifestyle factors: “Medical history includes [smoking status] smoking history and [alcohol status] alcohol use.” Categorical variables are expanded to their full clinical descriptions (e.g., “MICU” becomes “Medical Intensive Care Unit”), and abbreviations are systematically expanded on first occurrence to avoid ambiguity. Missing values are encoded as “not recorded” rather than being omitted, preserving information completeness.

The pipeline processes demographics (age, gender, race, etc.), admission details (type, location, unit, etc.), physical measurements (height, weight, BMI calculation), lifestyle factors (smoking, alcohol, drug abuse), clinical scores (GCS,^41^ SOFA,^43^ APACHE, etc.), and medical abbreviations (avoiding duplicate expansions). Finally, the datasets were divided equally into 75-12.5-12.5 train-validation-test formats for all downstream model training (including ALFIA and other comparable models).

#### ALFIA architecture

The proposed model architecture, ALFIA (Adaptive Layer Fusion with Intelligent Attention), is intended to efficiently use hierarchical features from pre-trained transformer models for text classification tasks (Figure 2). It has three primary sequential components: a Base Transformer Model, an Adaptive Layer Fusion (ALF) module, and an Attentional Classifier Head. Low-Rank Adaptation (LoRA) is an option for efficient fine-tuning.

#### Base transformer backbone

The foundation of the ALFIA architecture is a pre-trained transformer backbone, such as BERT,^44^ RoBERTa,^45^ BioBERT,^46^ or BioLinkBERT.^28^ This backbone serves as the primary feature extractor. Given an input sequence of tokens *X* = {*x*_1_,*x*_2_, …,*x*_*T*_}, where *T* is the sequence length, the Base Transformer Model outputs a series of hidden state sequences from its *L* layers:

$$H^{\left(0\right)},H^{\left(1\right)},\ldots ,H^{\left(L\right)}$$

Here, *H*^(0)^ represents the initial token and positional embeddings. For each layer *l*∈[1,*L*], $H^{\left(l\right)}=\left\{h_{1}^{\left(l\right)},h_{2}^{\left(l\right)},\ldots ,h_{T}^{\left(l\right)}\right\}$ is the sequence of hidden states, where each $h_{t}^{\left(l\right)}\in R^{d_{\text{model}}}$ is the hidden state for token *t* at layer *l*, and *d*_model_ is the dimensionality of the hidden states.

#### Adaptive layer fusion (ALF) module

The ALF module is a critical component designed to dynamically integrate information from multiple layers of the base transformer model. Its overall motivation is related to attention-based late fusion strategies for task adaptation,^47^ but it is tailored here for text-encoded structured EHR prediction. It takes as input the hidden states from the top *N*_*f*_ layers, i.e., $\left\{H^{\left(L-N_{f}+1\right)},\ldots ,H^{\left(L\right)}\right\}$. The objective is to learn a weighted combination of these layer representations, allowing the model to capture a richer set of features spanning different levels of abstraction.

#### The ALF module operates in several stages

##### Layer weight computation

This stage determines the importance (weight) *λ*_*j*_ for each of the *N*_*f*_ selected layers. First, an input layer summary *s*_*j*_ for each layer *j* (from the *N*_*f*_ layers) is computed via attention-mask-weighted average pooling of its token hidden states $h_{t}^{\left(j\right)}$:

$$s_{j}=\frac{\sum_{t=1}^{T}A_{t}\cdot h_{t}^{\left(j\right)}}{\sum_{t=1}^{T}A_{t}}$$

where *A*_*t*_ is the attention mask (*A*_*t*_ = 1 for real tokens, 0 for padding). These *N*_*f*_ summary vectors $\left\{s_{1},\ldots ,s_{N_{f}}\right\}$, each in $R^{d_{\text{model}}}$, are stacked into $S_{\text{stack}}\in R^{N_{f}\times d_{\text{model}}}$. Next, an inter-layer attention mechanism computes layer contributions. A global query vector $q_{\text{global}}=\text{mean}\left(s_{1},\ldots ,s_{N_{f}}\right)$ is formed. This query and *S*_stack_ are projected into Query (*Q*_proj_), Key (*K*_proj_), and Value (*V*_proj_) spaces:

$$\begin{array}{rr} Q_{\text{proj}} & =q_{\text{global}}W_{Q}\in R^{d_{k}\cdot n_{h}}\\ K_{\text{proj}} & =S_{\text{stack}}W_{K}\in R^{N_{f}\times \left(d_{k}\cdot n_{h}\right)}\\ V_{\text{proj}} & =S_{\text{stack}}W_{V}\in R^{N_{f}\times \left(d_{v}\cdot n_{h}\right)} \end{array}$$

where *W*_*Q*_,*W*_*K*_,*W*_*V*_ are trainable weight matrices, *n*_*h*_ is the number of attention heads, and *d*_*k*_,*d*_*v*_ are head dimensions. Attention scores are calculated as:

$$\begin{array}{rr} \text{scores}= & \text{softmax}\left(\frac{Q_{\text{proj}}K_{\text{proj}}^{T}}{\sqrt{d_{k}}}\right)\\ & \in R^{1\times N_{f}}\;\left(\text{per}\;\text{head}\right) \end{array}$$

The output context $c_{\text{layer}\_\text{attn}}=\text{scores}\cdot V_{\text{proj}}$ is projected to $c_{\text{layer}\_\text{attn}}^{\prime }=c_{\text{layer}\_\text{attn}}W_{O}\in R^{d_{\text{model}}}$. Optionally, if layer gating is enabled, these are passed through a linear layer and sigmoid:

$$\lambda =\text{sigmoid}\left(c_{\text{layer}\_\text{attn}}^{\prime }W_{\text{gate}}+b_{\text{gate}}\right)\in R^{N_{f}}$$

Otherwise, normalized attention scores are used as *λ*.

**Weighted Combination of Layer Hidden States:** The full hidden state sequences from the selected *N*_*f*_ layers are combined using the learned layer weights *λ*_*j*_. For each token position *t*:

$$h_{t}^{\text{fused}\_\text{raw}}=\sum_{j=1}^{N_{f}}\lambda_{j}\cdot h_{t}^{\left(L-N_{f}+j\right)}$$

This results in a sequence $H^{\text{fused}\_\text{raw}}\in R^{T\times d_{\text{model}}}$.

**Post-Fusion Processing:** The raw fused states $H^{\text{fused}\_\text{raw}}$ undergo further transformations, including Layer Interaction, Content Projection, and an Enhancement step:

$$\begin{array}{rr} H^{\text{enhanced}}= & {\text{LayerNorm}}_{1}(H^{\text{fused}\_\text{raw}}\\ & +H^{\text{projected}}+H^{\text{interaction}}) \end{array}$$

These operations typically involve sequences of Linear, Layer Normalization, GELU, and Dropout layers.

**Token-Level Attention for Local Context:** The enhanced states *H*^enhanced^ are processed by a token-level attention mechanism. Token scores score_*t*_ are computed for each token $h_{t}^{\text{enhanced}}$, masked, and softmaxed to yield token weights *β*_*t*_:

$$\beta_{t}={\left[\text{softmax}\left({\text{scores}}_{\text{masked}}\right)\right]}_{t}$$

The local context *c*_local_ is then a weighted sum

$$\begin{array}{rr} c_{\text{local}}= & \sum_{t=1}^{T}\beta_{t}\cdot h_{t}^{\text{enhanced}}\\ & \in R^{d_{\text{model}}} \end{array}$$

**Global Context Extraction:** A global context vector *c*_global_ is derived from *H*^enhanced^ via attention-mask-weighted average pooling, followed by a global context processing layer:

$$\begin{array}{rr} c_{\text{global}\_\text{pooled}} & =\frac{\sum_{t=1}^{T}A_{t}\cdot h_{t}^{\text{enhanced}}}{\sum_{t=1}^{T}A_{t}} \end{array}$$

$$\begin{array}{rr} c_{\text{global}}= & \text{GlobalContextLayer}\left(c_{\text{global}\_\text{pooled}}\right)\\ & \in R^{d_{\text{model}}} \end{array}$$

**Final Context Fusion and Output:** The local and global context vectors are concatenated, $\left[c_{\text{local}};c_{\text{global}}\right]\in R^{2\cdot d_{\text{model}}}$, and then fused by a Context Fusion layer to produce $c_{\text{fused}}\in R^{d_{\text{model}}}$. An output projection and LayerNorm yield the final ALF output vector $H_{\text{ALF}\_\text{out}}$:

$$\begin{array}{rr} H_{\text{ALF}\_\text{out}}= & {\text{LayerNorm}}_{2}(c_{\text{fused}}\\ & +\text{OutputProjection}\left(c_{\text{fused}}\right))\\ & \in R^{d_{\text{model}}} \end{array}$$

This vector $H_{\text{ALF}\_\text{out}}$ serves as the enriched representation for the subsequent classifier.

**Distinctive Advantages of ALF:** The ALF module distinguishes itself from traditional fusion strategies in two key aspects. First, unlike static pooling methods (e.g., mean/max pooling) or using only the final [CLS] token, ALF dynamically learns the importance weights *λ*_*j*_ for each layer. Since different layers in BERT-based models capture distinct linguistic and semantic features—ranging from surface-level syntax in lower layers to complex semantic dependencies in higher layers—our attention-based mechanism enables the model to adaptively emphasize the most relevant abstraction levels for each specific clinical input. Second, compared to concatenation-based fusion which linearly increases feature dimensionality with the number of layers, ALF maintains a compact representation space (*d*_model_) through weighted integration. This dimensionality preservation is crucial for preventing overfitting on limited clinical datasets while still enriching the feature representation with multi-scale information.

#### Attentional classifier head (ACH) module

The fused embedding $H_{\text{ALF}\_\text{out}}$ (denoted as *Z* for simplicity) from the ALF module is processed by the Attentional Classifier Head. First, *Z* is unsqueezed to $Z^{\prime }\in R^{1\times d_{\text{model}}}$ to be compatible with attention mechanisms expecting sequence input. It then undergoes multi-head self-attention:

$$\begin{array}{rr} \text{AttnOut}= & \text{MultiheadAttention}(Q=Z^{\prime },\\ & \;K=Z^{\prime },V=Z\prime ) \end{array}$$

This allows features within the fused representation *Z* to interact and be re-weighted. Following this, standard transformer block operations, including Layer Normalization, residual connections, and a FeedForward Network (FFN), are applied:

$$\begin{array}{rr} Z_{\text{norm}1}^{\prime }= & {\text{LayerNorm}}_{1}(Z^{\prime }\\ & +\text{Dropout}\left(\text{AttnOut}\right)) \end{array}$$

$$\begin{array}{cc} Z_{\text{ffn}}^{\prime } & =\text{FFN}\left(Z_{\text{norm}1}^{\prime }\right) \end{array}$$

$$\begin{array}{rr} Z_{\text{norm}2}^{\prime }= & {\text{LayerNorm}}_{2}(Z_{\text{norm}1}^{\prime }\\ & +\text{Dropout}\left(Z_{\text{ffn}}^{\prime }\right)) \end{array}$$

The processed vector $Z_{\text{norm}2}^{\prime }$ (squeezed back to $R^{d_{\text{model}}}$) is then passed to an output linear layer to produce the logits for classification:

$$\text{logits}=Z_{\text{norm}2}^{\prime }W_{\text{out}}+b_{\text{out}}$$

where *W*_out_ and *b*_out_ are the weight matrix and bias of the output layer, respectively. The logits are typically passed through a softmax function to obtain class probabilities.

#### Low-Rank Adaptation (LoRA)

To facilitate efficient fine-tuning, especially for large pre-trained models, Low-Rank Adaptation (LoRA) can be optionally integrated.^48^^,^^49^ When LoRA is enabled, for a pre-trained weight matrix $W_{0}\in R^{d_{1}\times d_{2}}$ within the Base Transformer Model (e.g., in attention or feedforward layers), its update Δ*W* is constrained to be of low rank. Specifically, *W*_0_ is kept frozen, and two trainable low-rank matrices, $A\in R^{d_{1}\times r}$ and $B\in R^{r\times d_{2}}$, are introduced, where the rank *r*≪*min*(*d*_1_,*d*_2_). The forward pass for a layer is modified from *h* = *W*_0_*x* to:

$$h=W_{0}x+BAx$$

During fine-tuning, only the parameters of matrices *A* and *B* are updated, significantly reducing the number of trainable parameters compared to full fine-tuning. This approach is based on the hypothesis that the adaptation of pre-trained models to new tasks often occurs in a low-rank subspace of the weight parameter space.

Algorithm 1 **Model Architecture Input: Raw text sequence T, Attention mask Mattn**

**Output**: Predicted class probabilities Pclass//Initialize Components.

1: BaseLM ← Pre-trained Transformer (e.g., BioBERT), optionally with LoRA.

2: FusionModule ← AdaptiveLayerFusion(Hsize, Nfuse, …).

3: ClassifierHead ← AttentionalClassifierHead(Hsize, Nclasses, …).

//Forward Pass through Base Language Model.

4: HiddenStatesall ← BaseLM.forward(T, Mattn) ⊳ Get all layer hidden states.

5: HiddenStatesfuse ← SelectTopLayers(HiddenStatesall, Nfuse).

//Adaptive Layer Fusion.

6: function AdaptiveLayerFusion.process (HSfuse, Mattn).

7: LayerRepresentations ← AveragePoolPerLayer(HSfuse, Mattn).

8: GateWeights ← CalculateLayerAttentionWeights(LayerRepresentations) ⊳ Via self-attention and optional gating.

9: WeightedStates ← *Σ* (GateWeights × HSfuse) ⊳ Element-wise product and sum.

10: EnhancedStates ← ProcessWeightedStates(WeightedStates) ⊳ Includes interaction, projection, LayerNorm.

11: LocalContext ← TokenAttentionPool(EnhancedStates, Mattn).

12: GlobalContext ← MaskedAveragePool(EnhancedStates, Mattn).

13: CombinedContext ← FuseContexts(LocalContext, GlobalContext) ⊳ e.g., Concat + Linear + Norm.

14: return CombinedContext, GateWeights.

15: FusedEmbed, LayerWeights ← FusionModule.process(HiddenStatesfuse, Mattn)//Attentional Classifier Head.

16: function AttentionalClassifierHead.process (FusedEmbed).

17: X ← SelfAttentionBlock(FusedEmbed.unsqueeze(1)) ⊳ MHA, Add & Norm, FFN, Add & Norm.

18: Logits ← OutputLinearLayer(X.squeeze(1)).

19: return Logits.

20: Logits ← ClassifierHead.process(FusedEmbed).

21: Pclass ← Sigmoid(Logits).

#### Model training, inference, and evaluation protocol

For all evaluations, we used the CW-24 MIMIC-IV subset as the primary benchmark and the eICU subset as the external validation cohort. ALFIA, its derivative models, and all comparison baselines were trained and evaluated through a unified pipeline. All scripts were assigned the same random seed of 42 to ensure reproducibility. For ALFIA, we designed training scripts capable of interfacing with encoded text inputs, leveraging validation set AUPRC for evaluation and early termination (default patience of 5 epochs). We integrated real-time monitoring of AUPRC and AUROC measurements for each epoch, with automatic storage of the best-performing LoRA Adapter, ALF, and ACH states. Final test set evaluation involved full computation of categorization metrics and 95% confidence intervals.

##### Expert evaluation protocol

To compare ALFIA’s performance with clinical expertise, we conducted an expert evaluation study involving two board-certified intensivists from the Department of Critical Care Medicine at Guangdong Medical University Affiliated Hospital, each with 5–10 years of ICU clinical experience. For this assessment, we randomly sampled 100 cases from each of the MIMIC-IV and eICU test sets (200 cases total). Experts were presented with the text-encoded clinical descriptions (identical to model input) and asked to predict mortality risk on a binary scale, along with their confidence level (low/medium/high). To ensure independence, experts evaluated cases without knowledge of actual outcomes or model predictions. We acknowledge that this sample size is relatively small and represents a limitation of the study; however, it provides preliminary evidence of model-clinician performance comparison. Future studies with larger expert panels and more cases would strengthen these findings.

For comparative baseline models, we employed the AutoGluon^34^ framework for unified training across a diverse ensemble of algorithms, including gradient boosting decision trees (LightGBM,^50^ XGBoost,^51^ CatBoost^52^), multi-layer neural networks (implemented using PyTorch^35^ and FastAI^53^), traditional machine learning models (k-nearest neighbors, random forest), and emerging transformer-based architectures (TabPFN,^19^ FT-Transformers^54^). All compared models use tabular data (encoded as text) as input, and utilize AUPRC as the primary training metric, with test set performance evaluated using identical classification metrics and 95% confidence interval estimates.

Algorithm 2 **Inference Pipeline Input: List of texts Stexts, Model configurations (LM name, paths to weights, Nfuse, Lmax), Batch size Bsize**

**Output**: DataFrame of fused text embeddings DFembed//1. Initialization.

1: BaseLM, FusionModule ← LoadModelsAndWeights(Model configurations).

2: BaseLM.eval(), FusionModule.eval().

3: Tokenizer ← LoadPretrainedTokenizer(LM name).

4: AllEmbeddings ← [], AllLayerWeights ← [].

//2. Process Texts in Batches.

5: for each batch of texts in Stexts do.

6: EncodedInput, Mattn ← TokenizeBatch(BatchTexts, Tokenizer, Lmax).

7: with torch.no_grad():

8: HiddenStatesall ← BaseLM.forward(EncodedInput[’input_ids’], Mattn).

9: HiddenStatesselect ← SelectTopLayers(HiddenStatesall, Nfuse).

10: if Nfuse >0 then.

11: FusedEmbedsbatch, Weightsbatch ← FusionModule(HiddenStatesselect, Mattn).

12: else.

13: FusedEmbedsbatch ← ZeroEmbeddingsForBatch().

14: Weightsbatch ← UniformWeightsForBatch().

15: Append FusedEmbedsbatch to AllEmbeddings.

16: Append Weightsbatch to AllLayerWeights.

//3. Finalize Output.

17: EmbeddingsArray ← VStack(AllEmbeddings).

18: LayerWeightsArray ← VStack(AllLayerWeights).

19: NormalizedEmbeddings ← Normalize(EmbeddingsArray).

20: DFembed ← CreateDataFrame(NormalizedEmbeddings, LayerWeightsArray).

For ALFIA derivative models (ALFIA-boost, ALFIA-nn), we exploited the embeddings created by ALFIA’s ALF module alongside original features as inputs to the AutoGluon model ensemble, serving as alternatives to the ALFIA ACH for prediction tasks. Classification metrics and 95% confidence intervals were obtained for all derivative model predictions.

### Quantification and statistical analysis

#### Performance metrics

Model performance was evaluated using Area Under the Precision-Recall Curve (AUPRC) as the primary metric due to class imbalance in mortality prediction (approximately 10% positive rate). Additional metrics included Area Under the Receiver Operating Characteristic Curve (AUROC), F1 score, and F2 score. All metrics were computed with 95% confidence intervals using bootstrap resampling (*n* = 1000 iterations).

#### Statistical significance testing

Pairwise comparisons between models were performed using Welch’s *t* test on bootstrap samples. *p*-values were adjusted for multiple comparisons using the Benjamini-Hochberg procedure. Statistical significance was defined as adjusted *p* < 0.05. Effect sizes were reported as relative improvement percentages. In figure legends where asterisks denote significance levels, the following convention is used throughout: ∗*p* < 0.05, ∗∗*p* < 0.01, ∗∗∗*p* < 0.001 (Welch’s *t* test with Benjamini-Hochberg correction; *n* = 1000 bootstrap iterations per model). The number of biological units (ICU admissions) for each dataset split is reported in the Results section and in the corresponding figure legends.

#### Hardware configuration

The experiments were carried out on cloud-based computing nodes with typical configurations. Each node included an NVIDIA RTX 4090 GPU with 24 GB of video RAM, a 16-core Intel Xeon(R) Platinum 8352V processor, and 120 GB of system memory. The nodes run Ubuntu 22.04 LTS, with GPU driver version 535.129.03 and CUDA support up to 12.6. All independent models were trained on a single GPU to maintain consistency in resource allocation and performance evaluation.

The Mamba package manager was used to manage the software environment, and Python 3.11.12 served as the base interpreter. Key packages include pandas 2.2.3 for data manipulation, numpy 1.26.4 for numerical computing, matplotlib 3.10.1 and seaborn 0.12.2 for data visualization, and scikit-learn 1.5.2 for machine learning utilities, torch 2.6.0 for deep learning framework support, transformers 4.49.0 for pre-trained model implementations, peft 0.15.2 for parameter-efficient fine-tuning, tqdm 4.67.1 for progress tracking, and AutoGluon 1.3.1 for automated batch machine learning. Portions of the statistical analysis and figure preparation were performed using GraphPad Prism version 10.4.2 software.
