# Supplementary material for: Predicting ICU in-hospital mortality from text-encoded structured EHR data using adaptive transformer layer fusion
Source: iScience. 2026 Jun 4;29(6):116135. doi: 10.1016/j.isci.2026.116135 (PMC13266190; doi:10.1016/j.isci.2026.116135)
Supplement: Document S1. Table S1 [file mmc1.pdf]

iScience, Volume *29*

Supplemental information

Predicting ICU in-hospital mortality from text-encoded structured EHR data using adaptive transformer layer fusion

Han Wang, Guoguang Lao, Ruoyun He, Ting Liu, Hejiao Luo, Changqi Qin, Hongying Luo, Yingqi Liu, Junmin Huang, Zihan Wei, Lu Chen, Yongzhi Xu, Ziqian Bi, Junhao Song, Tianyang Wang, Xin Liang Chia, Xuanhe Hou, Huafeng Liu, Junfeng Hao, and Chunjie Tian

Supplemental Table and Legend

Table S1. Variable domains and harmonized text-encoding rules used to generate the CW-24 clinical descriptions. The supplemental file summarizes the structured EHR domains explicitly described in the main manuscript, including demographic fields, admission context, anthropometrics, severity scores, dynamic assessments, and lifestyle or comorbidity indicators. Dynamic variables were summarized within the first 24 hours using maximum, minimum, first, last, mean, and standard deviation where available, and missing values were rendered as "not recorded" during text generation.

**Domain**	**Variables or fields**	**Encoding in the clinical description**	**Harmonization or derivation rule**
Demographics	Age, sex or gender, and race or ethnicity when available	Opening patient sentence (for example: age-year-old sex of ethnicity)	Direct cohort fields retained as harmonized descriptors across datasets
Admission context	ICU unit type, admission type or location, and first ICU or hospital stay flags	Admission clause describing where the patient was admitted	Categorical values expanded to full clinical names such as MICU to Medical Intensive Care Unit
Anthropometrics	Height, weight, and calculated body mass index	Physical examination sentence	BMI recalculated from height and weight when both are available
Severity scores	APACHE III or IV, SAPS II, OASIS, LODS, MELD, and SIRS when available	Clinical severity assessment sentence	Dataset-specific scores retained by name while preserving cohort provenance
Dynamic assessments	Glasgow Coma Scale and SOFA summaries within the first 24 hours	Severity assessment sentence using summary statistics	Maximum, minimum, first, last, mean, and standard deviation computed inside the 24 h prediction window

Domain	Variables or fields	Encoding in the clinical description	Harmonization or derivation rule
Lifestyle and comorbidity flags	Smoking status, alcohol abuse, drug abuse, and obesity indicators	Medical history sentence	Boolean or categorical indicators mapped to explicit natural-language statements
Missingness rule	Any structured field lacking a recorded value	Encoded as the phrase not recorded rather than omitted	Preserves absence information instead of silently dropping variables
